# Systemic Lupus Erythematosus in India: A Clinico-Serological Correlation

**DOI:** 10.7759/cureus.25763

**Published:** 2022-06-08

**Authors:** Rachita Mathur, Kirti Deo, Aishwarya Raheja

**Affiliations:** 1 Dermatology, Sawai Man Singh (SMS) Medical College, Jaipur, IND; 2 Dermatology, Dr. D.Y. (Dnyandeo Yashwantrao) Patil Medical College, Hospital & Research Centre, Dr. D.Y. Patil Vidyapeeth, Pune, IND

**Keywords:** sle, india, serology, autoantibodies, photosensitivity, malar rash, lupus

## Abstract

Background and objectives

Systemic lupus erythematosus (SLE) is a chronic multisystem disorder exhibiting a wide spectrum of clinical and immunological abnormalities. Skin is the second most affected organ; lesions may precede systemic manifestations and foretell systemic involvement. Correlation between systemic manifestations and immunological profile is known but the interplay between antibodies and cutaneous findings is an area of recent interest. The present study aims to evaluate the demographic differences, pattern and prevalence of skin lesions, and correlation between cutaneous, systemic manifestations, and serological profile in SLE.

Methods

A total of 40 patients diagnosed with SLE, fulfilling Systemic Lupus International Collaborating Clinics (SLICC) criteria (2012), who visited the Dermatology outpatient department between April 2019 to April 2020 were recruited. Demographic details, evaluation of cutaneous lesions as lupus erythematosus (LE) specific and LE non-specific, systemic examination, hematological tests, and serological profile findings were noted.

Results

The mean age of onset was 23.3 years with a female to male ratio of 19:1. Common LE-specific lesions were malar rash (77.5%), photosensitivity (70%), and generalized maculopapular rash (20%). Frequently occurring LE non-specific lesions were non-scarring alopecia (60%), oral ulcers (45%), and vasculitis (12.5%). Arthritis (77.5%) and nephritis (30%) were common systemic findings. Among 14 patients with cutaneous manifestations alone, 12 (85%) had antinuclear antibody (ANA), eight (57%) had anti-double-stranded DNA (anti-dsDNA), four (28%) had anti-Smith (anti-Sm) and anti-RO/Sjögren's syndrome antigen A (Anti-RO/SSA), three (21%) had anti-histone, and one (7%) had anti-ribonucleoprotein (anti-RNP) antibodies in serum.

Conclusions

Lower age at onset, high prevalence of photosensitivity, anemia, and alopecia with a low prevalence of Raynaud’s phenomenon suggest environmental influence in the context of the Indian population. A positive immunological profile in patients with cutaneous involvement alone gives an opportunity to the caregiver to identify the disease process much before systemic manifestations are expressed.

## Introduction

Systemic lupus erythematosus (SLE) is a chronic multisystem autoimmune disorder that exhibits a wide spectrum of clinical and immunological abnormalities ranging from localized cutaneous involvement to life-threatening systemic involvement [1,2].

The heterogeneity of SLE manifestations is due to an interplay of genetic, environmental, and hormonal influences [3]. The role of environmental influence is indicated by different manifestations in patients belonging to the same ethnicity and genetic ancestry but residing at different geographical locations [4,5].

Cutaneous involvement is as common as joint involvement [6] and 70-85% of the patients develop mucocutaneous manifestations at some point of time in the disease evolution [7], with 20-25% of patients presenting with cutaneous signs before systemic manifestations [8].

The cutaneous findings can be classified into lupus erythematosus (LE)-specific and LE non-specific lesions, according to Gilliam and Sontheimer, based on lesional histopathology [9]. In the Indian population, among LE-specific, the malar rash is most frequent, reported in 53.18% and 80% of patients by Ghosh et al. [10] and Vaidya et al. [11], respectively, followed by photosensitivity and diffuse maculopapular rash. Common non-specific lesions are non-scarring diffuse alopecia (86.67%), oral ulcers (56.67%), and Raynaud's phenomenon (6.67%) [10]. These lesions have a high sociomedical and socioeconomic impact due to the cost of management, physical disability, and vocational handicap.

Cutaneous manifestations represent the tip of an iceberg that constitutes life-threatening multiorgan involvement. Cutaneous findings and their alterations may point to a particular systemic involvement. Complete clinical evaluation including a serological profile in SLE patients is beneficial not just for diagnosis, but also for assessing disease activity (for example, using the SLE disease activity index 2000 (SLEDAI-2K)), interdisciplinary collaboration in treatment, and better prognosis.

## Materials and methods

Approval was taken from the Dr. D.Y. Patil Medical College, Hospital & Research Centre Institutional Ethics Sub-committee, Pune, India (Research Protocol No. I.E.S.C./FP/2018/40), and written informed consent was obtained from the patients. Male and female patients of SLE between 15-60 years of age, both treated and untreated cases, attending the Dermatology outpatient department of Dr. D.Y. Patil Medical College, Hospital and Research Centre, Pune, Maharashtra, between April 2019 to April 2020, were included in the study. Incidentally diagnosed cases were also enrolled in the same visit. Previously diagnosed cases of SLE, patients developing cutaneous manifestation for the first time, or patients visiting dermatology clinic for an existing cutaneous manifestation, were also included. The diagnosis of SLE was based on the fulfillment of the Systemic Lupus International Collaborating Clinics (SLICC) 2012 criteria [12]. The presence of four of the clinical and immunological criteria, including at least one clinical criterion, or biopsy-proven nephritis in presence of antinuclear antibodies (ANA) or anti-double-stranded DNA (anti-dsDNA) antibodies, was considered diagnostic of SLE. Patients who declined participation, did not fulfill SLICC criteria, or had comorbid skin diseases were excluded.

Demographic details like age, gender, and age of onset of SLE (cutaneous or systemic onset, whichever earlier) were recorded. Detailed evaluation of cutaneous lesions as LE specific and LE non-specific was done according to Gilliam and Sontheimer’s classification. Following a systemic evaluation, patients were also referred to the Rheumatology outpatient department. 

Laboratory investigations

Standard laboratory investigations included complete blood count, erythrocyte sedimentation rate, urine analysis, and C reactive protein. Serology was studied using a Euroline ANA Profile test (Euroimmun Medizinischa Labordiagnostika AG, Lübeck, Germany). ANA was detected by indirect immunofluorescence using Hep-2 as substrate, anti-dsDNA by antigen strips coated with dsDNA isolated from salmon testes, and anti-Sjögren's-syndrome antigen A (anti-SSA), anti-Sjögren's syndrome type B (anti-SSB), anti-Smith (anti-Sm), and anti-U1-ribonucleoprotein (U1-RNP) by affinity chromatography using bovine and rabbit thymus.

In patients with lesions mimicking other dermatoses or to diagnose a lesion as LE specific or non-specific, a skin biopsy was performed. The biopsy specimen for histopathological examination was formalin-fixed and stained with hematoxylin and eosin (H&E) and Alcian blue stains. Histological criteria for LE proposed by Bangert et al. were followed, which included five histologic features: hyperkeratosis, basement membrane thickening, follicular damage, leukocyte infiltration, and deep dermis involvement [13].

Statistical analysis

Statistical analysis was performed using IBM SPSS Statistics for Windows, Version 20.0 (Released 2011; IBM Corp., Armonk, New York, United States). Continuous variables were presented as measures of central tendency. Categorical numbers were presented as absolute numbers and percentages. For all statistical tests, a p-value less than 0.05 was taken to indicate significant difference.

## Results

During the study period, 40 patients were identified with SLE. The study group comprised 38 females (95%) and two males (5%) with a female:male ratio of 19:1. The patients' age range was 16-65 years with a mean age at presentation being 31.8 years. The most common age group was the third decade (50%). The age of onset was 13-56 years (mean age of onset 23.3 years) (Table 1). The youngest patient was a 16-year-old female who developed the disease at 13 years. All patients had skin lesions at the time of presentation and 26 (65%) patients had systemic manifestations as well.

**Table 1:** Demographic characteristics of the study group of 40 SLE patients SLE: systemic lupus erythematosus

Characteristic	Number	%
Sex		
Female	38	95
Male	2	5
Age (years)		
<20	6	15
21-30	20	50
31-40	6	15
>40	8	20
Age of onset (years)		
<20	12	30
20-30	21	52.5
30-40	4	10
>40	3	7.5

**Figure 1:**
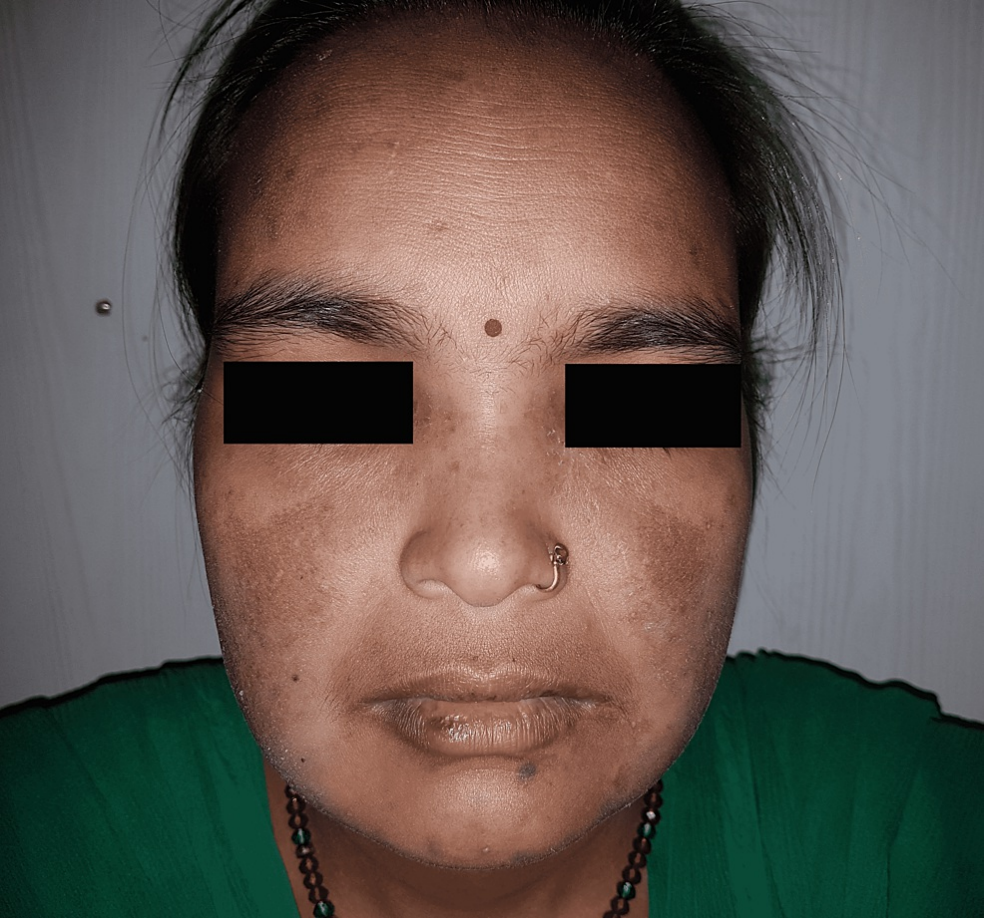
Malar rash in a 38-year-old SLE patient Malar rash or butterfly rash is characterized by an erythematous flat or raised rash across the bridge of the nose and cheeks, which usually spares nasolabial folds SLE: systemic lupus erythematosus

**Figure 2:**
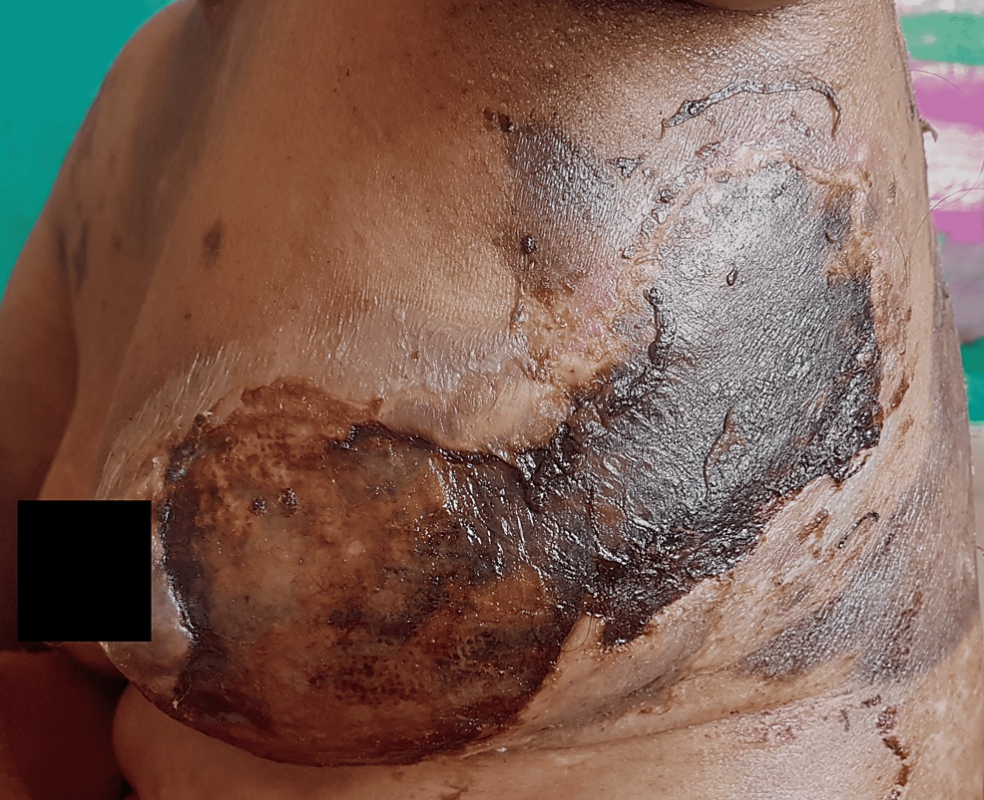
Epidermal detachment over left side of trunk representing TEN-like lesions in a 55-year-old female SLE patient TEN-like lupus is characterized by epidermal loss and mucosal ulceration occurring in patients with acute severe flares of SLE SLE: systemic lupus erythematosus; TEN: toxic epidermal necrolysis

The LE specific lesions noted were malar rash in 31 patients (77.5%) (Figure 1), photosensitivity in 28 patients (70%), generalized maculopapular rash in eight (20%), discoid rash in four (20%), subacute cutaneous lupus erythematosus (SCLE) in three (7.5%), and one patient (2.5%) each of lupus profundus and TEN-like SLE (Figure 2). Variants like lichenoid, mucosal, lupus tumidus, and chillblain lupus were not observed.

Among LE non-specific lesions, most commonly seen were non-scarring alopecia in 24 (60%), followed by oral ulcers in 18 (45%), Raynaud's phenomenon in four (10%), vasculitis in five (12.5%), bullous lesions in two (5%), and periungual telangiectasia, panniculitis, and erythema multiforme in one patient each. Livedo reticularis, sclerodactyly, calcinosis, pyoderma gangrenosum, leg ulcers, urticaria, erythromelalgia, and lichen planus were not observed. 

In systemic manifestations, polyarthritis was seen in 31 (77.5%), constitutional symptoms in 25 (62.5%), nephritis in 12 (30%), gastrointestinal involvement in nine (22.5%), cardiopulmonary in seven (17.5%), neuropsychiatric in five (12.5), and lymphadenopathy in four (10%) (Table 2).

**Table 2:** Prevalence of cutaneous (LE specific and LE non-specific) and systemic manifestations among 40 cases of SLE LE specific lesions are unique to SLE and their histology shows vacuolar interface dermatitis, which is diagnostic of SLE. LE non-specific lesions can be found in any connective tissue disorders and their histology is non-diagnostic. LE: lupus erythematosus; TEN: toxic epidermal necrolysis; SCLE: subacute cutaneous lupus erythematosus; SLE: systemic lupus erythematosus

S. No.	Clinical finding	Number of cases (n=40)	%
	Cutaneous manifestation:		
A.	LE specific		
1	Malar rash	31	77.5
2	Photosensitivity	28	70
3	Maculopapular rash	8	20
4	Discoid rash	4	10
5	TEN-like SLE	1	2.5
6	Hypertrophic LE	0	0
7	Lupus profundus	1	2.5
8	Mucosal lesions	0	0
9	LE Tumidus	0	0
10	Chilblain lupus	0	0
11	Lichenoid lesions	0	0
12	SCLE	3	7.5
B.	LE non-specific		
1	Non-scarring alopecia	24	60
2	Oral ulcer	18	45
3	Raynaud's phenomenon	4	10
4	Bullous lesion	2	5
5	Vasculopathy	0	0
6	Vasculitis	5	12.5
7	Erythema multiforme	1	2.5
8	Leg ulcer	0	0
9	Urticaria	0	0
10	Periungual telangiectasia	1	2.5
11	Panniculitis	1	2.5
	Systemic manifestation:		
1	Constitutional symptoms (Fever, myalgia, anorexia)	25	62.5
2	Lymphadenopathy	4	10
3	Arthritis	31	77.5
4	Nephritis	12	30
5	Cardiopulmonary manifestations	7	17.5
6	Neuropsychiatric manifestations	5	12.5
7	Gastrointestinal manifestations	9	22.5

Out of 12 patients with nephritis, 10 underwent kidney biopsy, which revealed minimal mesangial lupus nephritis (Class I) in one patient, mesangial proliferative lupus nephritis (Class II) in three patients, focal lupus nephritis (Class III) in two patients, and diffuse lupus nephritis (Class IV) in four patients. Biopsy could not be performed on two patients due to low blood cells count. One patient with cardiopulmonary involvement developed serositis in the form of massive pleural effusion with breathlessness and was revived by multiple sessions of pleural tapping.

Other systemic involvement included: three patients with neuropsychiatric involvement developed generalized tonic-clonic seizures, two patients had hypothyroidism, two patients developed avascular necrosis (AVN) of the head of the femur, and one of them underwent a total hip replacement.

One female patient who had fever, breathlessness, and arthritis, subsequently developed a rare presentation of TEN-like lesions, with sloughing of epidermis more marked over sun-exposed areas and necrotic patches on the trunk. 

Both the male patients had severe multisystem involvement. One presented with polyarthritis and nephritis (grade IV lupus nephritis), and the other developed anemia, polyarthritis, and serositis.

Laboratory investigations revealed that 32 patients (80%) had anemia, 11 (27.5%) had leucopenia, nine (22.5%) had thrombocytopenia, 15 (37.5%) had raised ESR, 11 (27.5%) had proteinuria, six (15%) had positive rheumatoid factor, and eight (20%) showed hypocomplementemia (C3,C4 levels). 

Overall, ANA were positive in 35 patients (85%), anti-dsDNA in 28 (75%), anti-Sm in 23 (57.5%), anti-RNP in nine (22.5%), anti-RO/SSA in 11 (27.5%), anti-histone antibodies in eight (20%), and anti-lupus anticoagulant in two patients (5%). Patients with cutaneous manifestations alone (35%), were also detected with serum antibodies: 12 (85%) had ANA, eight (57%) had anti-dsDNA, four (28%) had anti-Sm and anti-RO/SSA, three (21%) had anti-histone, and one (7%) had anti-RNP antibodies in serum. Patients with cutaneous as well as systemic manifestations (65%) were detected with anti-ANA in 23 (88%), anti-dsDNA in 20 (76%), anti-Sm in 19 (73%), anti-RNP in eight (30%), anti-RO/SSA in seven (26%), and anti-histone antibodies in five (19%) patients (Table 3).

**Table 3:** Autoantibodies profile of SLE patients and its correlation with cutaneous and systemic disease ab: antibody; ANA: antinuclear antibody; dsDNA: double-stranded DNA; Sm: Smith; RNP: ribonucleoprotein; SLE: systemic lupus erythematosus; SSA: Sjögren's syndrome antigen A

Positive antibodies	Overall no. of patients (%)	Patients with cutaneous involvement (%)	Patients with cutaneous and systemic involvement (%)
ANA ab	34 (85)	12 (85)	23 (88)
Anti-dsDNA ab	28 (70)	8 (57)	20 (76)
Anti-Sm ab	23 (57.5)	4 (28)	19 (73)
Anti-RO/SSA ab	11 (27.5)	4 (28)	7 (26)
Anti-histone ab	08 (20)	3 (21)	5 (19)
Anti-RNP ab	09 (22.5)	1 (7)	8 (30)

## Discussion

Over the years, various antibodies have been known to have a predictive value in the systemic profile of SLE patients. In this study, we have tried to make an attempt to correlate cutaneous manifestations with antibody profile as well as systemic involvement.

Our study reported female preponderance with a female to male ratio of 19:1 whereas Ghosh et al. and Malviya et al. reported a female to male ratio of 14:1 and 8:1, respectively [10,14]. Sex hormones are known to influence SLE, as estrogens are immune enhancing while androgens are immunosuppressive [15]. In the present study, the patients’ age range was 16-65 years, and the mean age for disease onset was 23.3 years, whereas it was 25 years in a study by Ghosh et al. and 24 years in a study by Malviya [10,14]. Lower age of onset has been reported among Indians and Southeast Asians and factors responsible for it are yet to be elucidated [4]. Adolescents generally present with a more severe illness than adults and accrue greater disease damage over time [16].

A total of 14 patients (35%) had cutaneous lesions as the initial presentation and were diagnosed as SLE on further evaluation in our study. Watson et al. reported cutaneous lesions as initial presentation in 25% of the cases [8].

Among LE specific lesions, the malar rash was the most common lesion observed in various studies [10,11]. Malar rash has been reported to be a marker of more severe systemic disease over time [17]. Photosensitivity is considerably common in the Indian population due to tropical location and global climatic change over the years. A study from southern India reported 52% and another from northern India reported 67% cases with photosensitivity [4]. Photosensitivity shows a strong association with the manifestation of all cutaneous LE subtypes, and the abnormal reactivity to ultraviolet (UV) light is involved in the pathogenesis of both cutaneous and systemic disease. A potentially crucial role in the initiation of autoimmune reaction cascade has been attributed to UV-induced keratinocyte apoptosis [18]. In Arabs, the majority of the studies show a high incidence of photosensitivity but few studies also observed a low incidence attributed to lower exposure to sunlight due to the traditional covering of the face [19,20]. The generalized maculopapular rash was observed in 20% of cases whereas it was considerably lower than reported by Wysenbeck (59%) [21]. It is an uncommon finding and is usually associated with previous sun exposure and involves sun-exposed areas [22]. The prevalence of discoid rash was similar to the study of Ghosh et al. (20%) and Kapadia [10,23].

**Figure 3:**
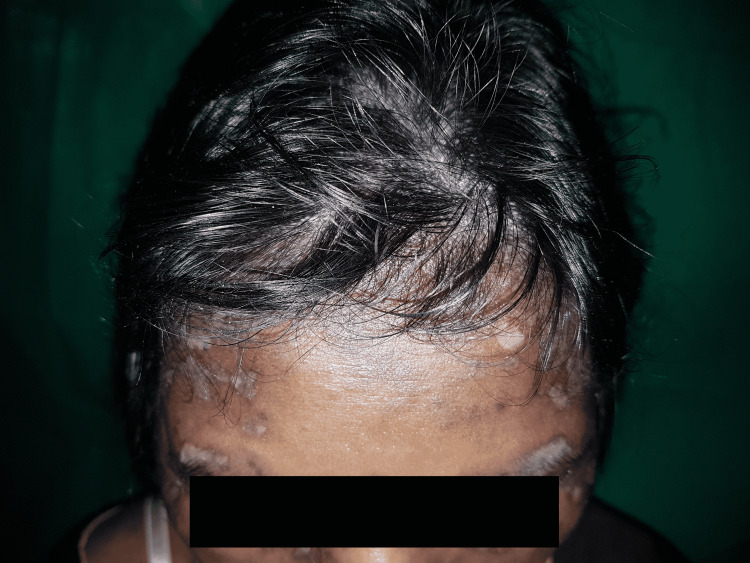
Lupus hair with discoid lupus erythematous lesions on scalp in a 35-year"old female with SLE Short and stubby frontal hair in SLE patients is referred to as "lupus hair", which occurs in about 30% of the patients. SLE: systemic lupus erythematosus

Among LE non-specific lesions, non-scarring alopecia was the most common finding (60%) as also reported by Malviya (80%) [14] and Maheshwari (80%) [15]; the presence of which may be contributed by the high prevalence of anemia in young Indian females. One commonly observed finding is short and stubby frontal hair, referred to as "lupus hair", which occurs in about 30% of SLE patients [24] (Figure 3).

We observed a similar prevalence of oral ulcers as reported by Malviya and Ghosh [10,14]. Raynaud's phenomenon is relatively uncommon in the Indian population. This may be due to high average temperatures in tropical areas of India.

TEN is a rare presentation in SLE with less than 10 cases reported worldwide. The term acute syndrome of apoptotic pan-epidermolysis (ASAP) has been proposed for the TEN-like cutaneous injury pattern. A similar injury pattern has been proposed in acute graft versus host disease, pseudoporphyria, and the classic drug-hypersensitivity syndrome [25]. Fas-Fas ligand interactions have been implicated in the massive keratinocyte apoptosis. Differentiating TEN-like cutaneous LE from drug-induced TEN is a common dilemma; the former has significant systemic disease activity (e.g., lupus nephritis or cerebritis), which requires prompt intravenous immunoglobulin and/or systemic corticosteroids.

Arthritis is usually the most frequent finding among systemic involvement. A non-erosive inflammatory rheumatoid arthritis such as polyarthritis is the commonest type, 10% of which can result in deformities and limitation of joint function [4]. Of cases in the present study, 30% were diagnosed as lupus nephritis and biopsy revealed its different classes as per the International Society of Nephrology classification of lupus nephritis. Various Indian studies show equal proportions of different classes of lupus nephritis except for the unusually high prevalence of membranoproliferative lesions in patients in western India [11,26]. Similarly, our study also revealed an almost equal incidence of all classes with a slightly higher proportion of diffuse lupus nephritis. Higher prevalence rates of neurological involvement have been reported in studies from western India [11,26]. The most common presentations are neuropsychiatric disorders, seizures, psychosis, and focal changes in the brain. Among cardiopulmonary findings, pericardial effusion, myocarditis, pulmonary hypertension, and valvular heart disease are common [4]. Pulmonary manifestations commonly include pleural effusion, interstitial lung disease, small lung, interstitial pneumonitis, and infections. Gastrointestinal symptoms are common; nausea and vomiting are the frequent findings followed by elevated liver enzymes, diarrhea, and anorexia. Almost two-thirds of the patients complain of constitutional symptoms, which include low-grade fever, fatigue, malaise, and a general feeling of being unwell [4,10]. Other uncommonly reported manifestations are ophthalmic, obstetric, and menstrual irregularities [18].

Anemia was observed in 80% of our cases, much higher than reported in previous studies (28.5%) [11]. The high prevalence may be due to dietary deficiency, poor socioeconomic status, and high prevalence in women of reproductive age group.

Comparing disease characteristics among the same ethnic population over a period of time gives an insight into its changing trends. Table 4 shows a comparison of disease characteristics in various Indian studies.

**Table 4:** Comparison of disease characteristics in various Indian studies ab: antibody; TEN: toxic epidermal necrolysis; ANA: antinuclear antibody; dsDNA: double-stranded DNA; Sm: Smith; SSA: Sjögren's syndrome antigen A

Manifestation	Malaviya et al., 1997 [4]	Vaidya et al., [11]	Ghosh et al., 2009 [10]	Maheshwari et al., 2017 [15]	Present study, 2019
Malar rash (%)	70	53.18	80	66.36	77.5
Photosensitivity (%)	48	9.55	50	66	70
Alopecia (%)	83	-	86.67	80	60
Oral ulcers (%)	55	-	56.67	70	45
Raynaud's phenomenon (%)	13.3	15.5	6.67	10	10
Bullous/TEN-like (%)	-	-	10	-	7.5
Renal (%)	57	-	46.67	43.36	30
Musculoskeletal (%)	85	-	90	86.36	77.5
Cardiopulmonary (%)	22	-	13.34	13.63	17.5
Neuropsychiatric (%)	51	-	73.34	42.72	12.5
ANA ab (%)	97	-	100	100	85
Anti-dsDNA ab (%)	68	-	83.34	60	70
Anti-Sm ab (%)	29	-	-	-	57.5
Anti-RO/SSA ab (%)	34	-	-	-	27.5

SLE being an autoimmune disease, over 100 different self-molecules have been known to bind autoantibodies; specifically, ANA, anti-dsDNA, anti-Sm, and antiphospholipid antibodies (APLA) are among the 11 criteria used for diagnosing SLE according to SLICC. The antibody pool of SLE patients, and hence their adaptive immune systems, may be fundamentally different; using these antibody repertoires healthy individuals can be separated from SLE patients in remission [27]. Not just limited to diagnosis, these antibodies are a harbinger of further disease activity.

ANAs can identify particular subsets of LE; anti-dsDNA is associated with renal involvement, anti-RO/SSA with SCLE rash, serositis, and hematological manifestations, anti-ribosomal P protein with neuropsychiatric disorders, and anti-RNP with arthritis, Raynaud’s phenomenon, and puffy fingers [28]. Shrivastava and Khanna proposed the cluster theory: distinct autoantibody clustering correlates to particular clinical presentation [29]. Cluster 1 (anti-Sm and anti-RNP) has the lowest incidence of proteinuria, anemia, lymphopenia, and thrombocythemia. Cluster 2 (anti-dsDNA, anti-RO/SSA, and anti-La) has a higher rate of nephritic syndrome and leukopenia. Cluster 3 (anti-ds-DNA, lupus anticoagulant, and anti-cardiolipin) is associated with thrombotic events. In our study, patients with cutaneous manifestations alone (35%) were also detected with serum antibodies; 12 (85%) had ANA, eight (57%) had anti-dsDNA, four (28%) had anti-Sm and anti-RO/SSA, three (21%) had anti-histone, and one (7%) had anti-RNP antibodies in serum. Similarly, in another study, patients with cutaneous involvement had positive ANA (65.1%), anti-dsDNA (60.4%), anti-Ro (51.2%), anti-Sm (37.2%), and anti-RNP (30.2%) antibodies [30]. Thus, the presence of this antibody pool foretells systemic involvement, giving the opportunity to the dermatologist to identify the disease process much before systemic manifestations are expressed.

A limitation of this study was the smaller study group with a shorter duration of follow-up. Also, histopathological correlation, immunofluorescence, periodic serological profile, direct Coombs test, and chemical analysis of urine could not be performed for every patient.

## Conclusions

Lower age at onset, high prevalence of photosensitivity, anemia, and alopecia with a low prevalence of Raynaud’s phenomenon suggests that there may be a role of environmental factors (pertaining to the Indian population in this report) in determining disease manifestations. Patients with cutaneous manifestations alone may have detectable serum antibodies. Relation between the presence of an antibody associated with systemic involvement has been established before; our study brings the focus to the presence of these antibodies in absence of any organ involvement other than skin. This provides a warning signal for predisposition to develop multiorgan manifestations in the near future. Cutaneous manifestations of SLE demand serological profile support not just for diagnosis but also for better prognosis.
